# Nutritional behavior and attitudes in food allergic children and their mothers

**DOI:** 10.1186/2045-7022-3-41

**Published:** 2013-12-10

**Authors:** Laura Polloni, Alice Toniolo, Francesca Lazzarotto, Ileana Baldi, Francesca Foltran, Dario Gregori, Antonella Muraro

**Affiliations:** 1 Referral Centre for Food Allergy Diagnosis and Treatment, Veneto Region, Department of Women and Child Health, Padua University Hospital, via Giustiniani 3, 35128 Padua, Italy; 2 Unit of Biostatistics, Epidemiology and Public Health, Department of Cardiac, Thoracic and Vascular Sciences, University of Padova, Padova, Italy

**Keywords:** Food allergy, Diet, Nutrition, Social impact, Children

## Abstract

**Background:**

Avoidance of food allergens requires adapting dietetic habits, changing nutritional approach. A restriction of food choice can result in a monotonous diet and impact social life. This study investigated the impact of food allergy on nutritional behavior and attitudes of patients and their families.

**Methods:**

A survey involving mothers of food allergic children aged 0–16 years was carried out. We primarily studied the variables related to the child (age, gender, clinical history, food and social events attitudes). In addition, Spielberg Trait-Anxiety Inventory (STAI-T) test was applied to the mothers. We assessed separately the associations between characteristics of child-mother pairs and diet monotony, and attendance to social events, by means of proportional odds regression models.

**Results:**

Nearly 10% of the 124 participants completely banned allergenic foods at home and 15.3% consumed their meals separately. More than one fourth attended parties rarely or never. Most of the participants reported a “monotonous diet”. Model results suggested significant associations between child age (p = 0.05), mother age (p = 0.05), number of excluded foods (p = 0.003) and monotony of the diet. The attendance of social events was inversely associated with the number of excluded foods (p = 0.04) and the mother’s STAI-T T-score (p = 0.04).

**Conclusions:**

The results highlighted the impact of food allergy in reducing interest about food and influencing patients’ approach to social life. It is important to support families in managing allergens avoidance.

## Introduction

Food allergy is a common disease, especially among children, with data suggesting a cumulative prevalence of 3% to 6% and a worrying increase in its incidence [1,2]. The only therapy is still the total avoidance of food allergens via elimination diets and emergency treatment of symptoms caused by unintentional ingestion. The limits imposed by the strict diet and the potential risk of anaphylaxis make food allergy a burden for patients and their families, affecting considerably their quality of life (QoL) and psychosocial well-being [3,4]. Childhood food allergy is demonstrated to have a significant impact on general health perception, emotional impact and limitation on family activities [5-7]. Avoidance of the implicated food requires families to adapt recipes and make appropriate substitutions: each family must determinate suitable approaches to best manage food allergies, varying as the child age or as the situation changes [8,9]. Attending birthday parties or visiting relatives requires of opportune strategies to ensure food allergic child’s safety [8]. Children who have experienced a severe reaction sometimes develop disordered eating or become withdrawn and fearful [10]. Only discussing food can generate anxiety in these patients and is often associated with physical reactions and illness [11]. Peanut allergic children are demonstrated to have more fear of adverse events and more anxiety about eating than diabetic children [12].

Studies on caregivers of food allergic children confirmed that food allergy significantly affects meal preparation and family social activities, reporting also a considerable impact on parents’ stress levels [13,14]. Mothers showed worse psychological and physical quality of life and higher anxiety and stress than fathers [15].

The present study was aimed to investigate the impact of food allergy on behavior and attitudes towards food among patients and their family. Particular attention was given to the influence of age, gender, clinical history and mother’s anxiety in the way of dealing with food allergy day by day.

## Methods

### Setting and sample

A survey about nutritional behavior and attitudes was carried out at the Referral Centre for Food Allergy Diagnosis and Treatment, Veneto Region in Padua (North Eastern Italy). Mothers were invited to participate while accompanying the children to clinical visits. They were given an information sheet outlining the study and encouraged to ask the researcher questions. If interested in participating, they signed a written consent form and provided their contact and demographic details as well as information about children clinical history. Then the survey was administered: it was completed by mothers only for pre-school children and by mothers and children together for patients older than 6 years old. On average this took approximately 20–25 min. Patients and their mothers were recruited over a 6 month period. Inclusion criteria included that children were confirmed suffering from immunoglobulin E (IgE)-mediated food allergy by an allergology and immunology specialist on the basis of a clinical history with evidence of sensitization and a positive food challenge or positive skin prick test and/or serum-specific IgE results. All participants developed food allergy in early childhood and did not suffer from serious concomitant non-allergic disease; children who did not complete weaning were excluded. Food involved in patients’ food allergy were milk, egg, wheat and nuts. Patients’ characteristics (Table 1) were assessed through a cross check between mothers report and case history available at the Centre.

**Table 1:** Patients’ and mothers’ characteristics

***Patients’ characteristics***	***N***	***(%)***	***Mean (SD)***
*Gender*			
Male	80	(64.5)	
Female	44	(35.5)	
*Age* 6.5 (4.1)			
0-5 years old	72	(59.1)	
6-11	34	(27.4)	
12-16	18	(14.5)	
Number of excluded foods			
1	44	(35.5)	
2	41	(33.1)	
>2	39	(31.4)	
*Previous anaphylaxis*	32	(25.8)	
*Adrenaline prescription*	60	(48.4)	
**Mothers’ characteristics**	**N**	**(%)**	**Mean (SD)**
*Age*			
18-39	76	(61.3)	
40+	48	(38.7)	
*T-score STAI-T*			46.8 (8.5)

The study was performed in respect of the Italian regulation regarding potential sensitive data and according to World Medical Association Declaration of Helsinki Ethical Principles for Medical Research Involving Human Subjects.

### Instruments

A questionnaire was created by a specialist in clinical psychology with expertise in food allergy, a dietician and a paediatric allergist. The survey consists of closed-ended questions assessing the presence of allergens at home, meal sharing, involvement in social activities including food and the approach towards food (monotony of the diet and interest about tasting new foods). A copy is available on request.

The STAI-T – Y form [16,17] was used to assess mothers’ trait mood through self report individual administration. Specific instructions are provided to the respondent accordingly to the test manual [16].

This simple, widely and worldwide used test consists of 20 questions requiring the subjects to describe how they generally feel and if their general response to situations is perceived as threatening to measure anxiety.

A norm-based T-score using the mean and standard deviation of the reference normative Italian population [16] has been calculated for each subject according to McCall formula. The mean of 50 represents the mean score of the general population and 10 the standard deviation. The mean T-score of the study sample can therefore be interpreted in terms of how many standard deviation units it is away from 50.

### Statistical analysis

Descriptive analysis was used to outline the characteristics of the participants and to illustrate the presence of allergens at home, meal sharing, involvement in social activities including food and the approach towards food.

By using ± 1 standard deviation from the mean of 50 as cut-off points for T-score STAI-T, three groups were defined as follows: women with score below 40 (low anxiety), women with scores from 40 to 60 (moderate anxiety) and women with scores higher than 60 (severe anxiety).

A proportional odds regression model for ordinal data was applied to each item score (dependent variable) and included child (gender, age, adrenaline prescription, previous anaphylactic reactions, number of excluded foods) and mother characteristics (age, categorized T-score STAI-T) as independent variables. This model is a generalization of ordinary logistic regression for binary outcomes and the results are expressed as Odds Ratio (OR) and 95% Confidence Interval (95% CI). Each OR may be interpreted as the effect of the variable on the odds of being in a higher category of the outcome (for monotonous diet score, the higher the worse; for party attendance score, the higher the better) across the entire range of values it takes.

Findings were analyzed using the STATA v9 statistical software package. The level of significance was set at 5%.

## Results

A total of 124 mother-child pairs consented to participate in the study. Most children were male, 0–5 years old and with a diet excluding more than one food item (Table 1).

Mothers’ mean level of anxiety was within the “normal” interval (mean T-score STAI-T 46.8, 95% CI: 45.3 - 48.3).

The findings show up that 9.8% (n = 13) of the families decided to absolutely exclude food allergens from their home. 15.3% (n = 19) of the patients consume their meal separately, not sharing it with other family member at home or peers in school canteens. Concerning attendance to social occasions involving foods, 44.3% (n = 55) declared to participate always, the 29% (n = 36) only sometimes, 21% (n = 26) rarely and 5.7% (n = 7) asserted they never attend to parties. Those who join in social gathering reported (only for participants older than 6 years old) they usually bring foods from home (n = 27) or eat only “safe foods” (reading labels) (n = 37), or take on both solutions; only 2 persons declared to not assume any kind of food when they attend to parties. Regarding interest in tasting new foods, in a rising rating likert scale from 1 to 5, the patients scored a mean of 3.3 (Median 3; Iqr 3). Most of the participants (n = 77; 62%) claimed to have a “monotonous diet”: in a rising rating scale from 1 to 5 they reported a mean score of 2.55 (Median 2; Iqr 3). When asked about causes of repetitive diet, they chose answers as follows: strict avoidance (n = 37), low curiosity about food (n = 30), a limited choice of food industry safe products (n = 23) and difficulties in making traditional recipes (n = 22).

The proportional odds regression model suggested significant associations between child age, mother age, number of excluded foods and monotony of the diet (Table 2). The more the child age increased the less the diet was monotonous (per unit increase of age OR = 0.90 and 95% CI: 0.82-0.99); on the other hand, the more the mother age raised the more the monotony of the feeding enhanced (40 or more vs. 18–39 years old OR = 2.13 and 95% CI: 0.99-4.62). An increase in the number of excluded foods was related to an augment of the repetitiveness of the diet (2 food items vs. 1 OR = 2.55 and 95% CI: 1.07-6.06; >2 food items vs. 1 OR = 4.71 and 95% CI: 1.94-11.4).

**Table 2:** Results of the ordinal logistic models

	***Monotony of the diet***			***Attendance to parties***		
**Participants’ characteristics**	**OR**	**95% CI**	**p-value**	**OR**	**95% CI**	**p-value**
*Gender*						
Male	1			1		
Female	0.62	0.31-1.27	0.19	1.52	0.71-3.25	0.28
*Age (continuous)*	**0.90**	**0.82-0.99**	**0.05**	1.10	0.99-1.23	0.07
*Number of excluded foods*						
1	1					
2	**2.55**	**1.07-6.06**	**0.03**	**0.32**	**0.13-0.78**	**0.01**
>2	**4.71**	**1.94-11.4**	**0.01**	0.57	0.23-1.40	0.22
*Previuous anaphylaxis*	0.58	0.26-1.30	0.19	0.68	0.30-1.54	0.35
*Adrenaline prescription*	1.34	0.60-2.97	0.47	0.78	0.34-1.82	0.57
*Mother’s age*						
18-39	1			1		
40+	**2.13**	**0.99-4.62**	**0.05**	0.62	0.28-1.37	0.24
*T-score STAI-T*						
<40	1			1		
40-60	0.85	0.36-2.01	0.72	**0.32**	**0.12-0.85**	**0.02**
≥60	0.29	0.06-1.29	0.10	0.72	0.15-3.37	0.68

Bold text highlights statistically significant results (p<.05).

The attendance to social events involving foods was found to decrease with the number of excluded foods (2 food items vs. 1 OR = 0.32 and 95% CI: 0.13-0.78) and the mother’s T-score STAI-T (moderate score vs. low score OR = 0.32 and 95% CI: 0.12-0.85).

## Discussion and conclusions

Even if morbidity and mortality from food allergy in children are generally low, strong evidence testifies that food allergy has a relevant impact on psychological distress and QoL of children and adolescents, as well as their families [3].

Since recently health related QoL has been recognized as an important outcome measure in clinical studies [18], new instruments for assessing QoL in food allergic children have been developed and validated in order to provide further insights into the problems these children encounter [3].

However, even if it is impossible to disregard that eating is strongly related with familial, social and group activities, the effects of food allergy on nutritional approach of patients and their families are still relatively under-explored.

The main objective of the present survey was verifying in a sample of mother-child pairs the impact of food allergy on family’s attitudes towards food, with implications for social life. According to our results, children food allergy seems to strongly influence the family eating habits of a portion of respondents: nearly 10% of the participants’ families decided to completely exclude food allergens from their home: this means that all family members follow the restricted diet; this is a “drastic” measure finalized to guarantee a safe environment to the allergic child, eliminating accidental ingestions and contaminations. However, excluding the offending food from the domestic setting could prevent the child from being trained to avoid allergens. In fact, families that bring the allergen into the home implement strategies to correctly discriminate between safe and not safe foods and have the opportunity to teach the child how to manage avoidance [8].

Moreover, even if most of the participants share meals with family or peers, about one sixth of the interviewed patients consumed their meals separately from the other family members and/or did not attend school canteens to minimize the risk of contaminations. This obviously can have an impact on social life [19,20]. This happens, above all, concerning attendance to social occasions involving foods: more than one fourth of the participants asserted they attend parties rarely or never. Particularly, in our sample, the increasing of the number of excluded foods was related to a decreasing on the attendance to social events. The result is in line with literature data reporting a significant disruption in family social events [3,7]: many parents would rather reduce the risk and concern induced by social activities by avoiding them altogether [13] and a number of parents report preventing their child from attending parties and school trips [21]. Interestingly the mothers’ trait anxiety T-score was found to impact on the attendance to social events involving foods, a moderate score, rather than a low score, being linked to a decreasing in joining social gathering.

The participants (older than 6 years old) who declared to attend parties always or sometimes reported to usually use two main strategies to cope with the burden of food allergy: many of them carefully check labels to assure the avoidance of allergens from foods, confirming how label reading represents the keystone of food allergy management [8,22,23]. As an alternative, children bring foods from home. Only in a very small number of cases participants reported to usually not assume any kind of food when attending social events.

A varied nutrient intake provides the opportunity for an adequate nutrient intake balance reducing malnutrition risks [24,25]. When asked about interest in tasting new foods and monotony of the diet, the participants showed a medium-level score and reported “strict avoidance” and “low curiosity about food” as the main causes of repetitive diet. This could be linked to the fact that children suffering from food allergy sometimes develop disordered eating or become withdrawn and fearful about food [10,11,26]. The avoidance of allergen traces is often a necessary strategy to stay away from the risk of reactions, however, it is found to be a heavy burden for patients and their families. A better allergy-specific QoL in mothers and their children who report eating products labelled “may contain nuts” than those who strictly avoided all nuts was reported [5]. In addition, many families reported that the most significant obstacle which prevents them leading a normal life is the widespread use of “allergen traces” labelling on pre-packed foods [23]. Other reasons reported in the present study for monotonous diet are, in fact, a limited choice of food industry safe products and difficulties in making traditional recipes.

The monotony of the diet was resulted influenced by the child and mother age in an opposite way. The more the mother age raised the more the monotony of feeding enhanced. Avoidance of the allergen requires mothers to learn to adapt recipes and make appropriate substitutions [8] so that more than 60% of caregivers reported that food allergy significantly affected meal preparation [13]. It could be that older mothers have more difficulties in making traditional recipes using alternative ingredients or in finding new adequate recipes. To the contrary, an increasing of the child age was linked to a decreasing on the repetitiveness of the diet: it could reflect children overcoming some food allergies [1] extend their diet, or simply change in dealing with food over time.

Similarly to what observed for the attendance to social events, the increasing of the number of excluded foods was related to an augment of the repetitiveness of the diet, confirming that the number of food allergies had a significant negative impact on family activities [13,27] and on the perceived overall health-related QoL [7].

Even if not as a primary intent, this study investigated also the influence of mothers’ trait anxiety on the food approach and management. Most of the patients’ mothers (n = 114; 91.9%) reported a moderate or low mean score when compared with the Italian normative data. At the contrary, some studies reported higher trait-anxiety levels in food allergic mothers than in norm means [5,15]. A possible explanation could be that all the participants of the present study attended a Referral Centre for Food Allergy Diagnosis and Treatment, where they received adequate information and support about the disease: this could have influenced mothers’ level of anxiety; therefore, caution is needed in generalizing these results. The mothers’ trait anxiety T-score was found to impact on the attendance to social events involving foods: a moderate score, rather than a low score, is linked to a decreasing in joining social gathering. A phenomenological study underlined how the feeling of “living with risk” was present in food allergic children’s mothers and it was associated to an emerging feeling of “living with fear” that could influence everyday life [28]. A link between mother’s wellbeing and “social” managing of food allergy was found; nevertheless further researches are needed to understand the underlying psychological mechanisms.

This study investigated the impact of food allergy on nutritional behavior and attitudes of the patients and their families, exploring the influence of some variables in their way of dealing with the disease. Gender and clinical history seemed to not influence the findings.

As far as we know, this is the first study focusing specifically on the issue. Our results underline the impact of food allergy in reducing interest about food and in influencing patients’ approach to social life: these findings stress in fact the importance of supporting families in managing psychosocial aspect of food avoidance and in arousing curiosity in children, suggesting recipes for a varied and stimulating diet.

## Abbreviations

CI: Confidence interval; IgE: Immunoglobulin E; OR: Odds ratio; QoL: Quality of life; STAI-T: Spielberg trait- anxiety inventory.

## Competing interests

No financial support was awarded relating to this paper. The authors declare that they have no competing interests.

## Authors’ contributions

LP designed the study, collected and analyzed the data and prepared the manuscript. AT and FL collected the data, provided intellectual input and assisted in manuscript preparation. IB and FF performed the analysis and prepared the manuscript. DG assisted and supervised the analysis. AM coordinated and supervised the study, provided intellectual input and assisted in manuscript preparation. All authors read and approved the final manuscript.
